# Stress-Induced Phosphorylation of Nuclear YB-1 Depends on Nuclear Trafficking of p90 Ribosomal S6 Kinase

**DOI:** 10.3390/ijms19082441

**Published:** 2018-08-18

**Authors:** Aadhya Tiwari, Simone Rebholz, Eva Maier, Mozhgan Dehghan Harati, Daniel Zips, Christine Sers, H. Peter Rodemann, Mahmoud Toulany

**Affiliations:** 1 Division of Radiobiology & Molecular Environmental Research, Department of Radiation Oncology, University of Tuebingen, Tuebingen, Germany; aadhya.tiwari@uni-tuebingen.de (A.T.); simone.rebholz@klinikum.uni-tuebingen.de (S.R.); eva.maier@student.uni-tuebingen.de (E.M.); mozhgan.dehghan-harati@klinikum.uni-tuebingen.de (M.D.H.); daniel.zips@med.uni-tuebingen.de (D.Z.); hans-peter.rodemann@uni-tuebingen.de (H.P.R.); 2 German Consortium for Translational Cancer Research (DKTK), Partner Site Tuebingen and German Cancer Research Center (DKFZ), Heidelberg, Germany; 3 Laboratory of Molecular Tumor Pathology and Systems Biology, Institute of Pathology, Charité Universitätsmedizin Berlin, Berlin, Germany; christine.sers@charite.de; 4 German Consortium for Translational Cancer Research (DKTK), Partner Site Berlin and German Cancer Research Center (DKFZ), Heidelberg, Germany

**Keywords:** ionizing radiation, EGF, oncogenic KRAS, nuclear YB-1, MAPK/ERK

## Abstract

Ionizing radiation (IR) and epidermal growth factor (EGF) stimulate Y-box binding protein-1 (YB-1) phosphorylation at Ser-102 in KRAS wild-type (KRASwt) cells, whereas in KRAS mutated (KRASmut) cells, YB-1 is constitutively phosphorylated, independent of IR or EGF. YB-1 activity stimulates the repair of IR-induced DNA double-strand breaks (DSBs) in the nucleus. Thus far, the YB-1 nuclear translocation pattern after cell exposure to various cellular stressors is not clear. In the present study, we investigated the pattern of YB-1 phosphorylation and its possible translocation to the nucleus in KRASwt cells after exposure to IR, EGF treatment, and conditional expression of mutated KRAS(G12V). IR, EGF, and conditional KRAS(G12V) expression induced YB-1 phosphorylation in both the cytoplasmic and nuclear fractions of KRASwt cells. None of the stimuli induced YB-1 nuclear translocation, while p90 ribosomal s6 kinase (RSK) translocation was enhanced in KRASwt cells after any of the stimuli. EGF-induced RSK translocation to the nucleus and nuclear YB-1 phosphorylation were completely blocked by the EGF receptor kinase inhibitor erlotinib. Likewise, RSK inhibition blocked RSK nuclear translocation and nuclear YB-1 phosphorylation after irradiation and KRAS(G12V) overexpression. In summary, acute stimulation of YB-1 phosphorylation does not lead to YB-1 translocation from the cytoplasm to the nucleus. Rather, irradiation, EGF treatment, or KRAS(G12V) overexpression induces RSK activation, leading to its translocation to the nucleus, where it activates already-existing nuclear YB-1. Our novel finding illuminates the signaling pathways involved in nuclear YB-1 phosphorylation and provides a rationale for designing appropriate targeting strategies to block YB-1 in oncology as well as in radiation oncology.

## 1. Introduction

Y-box binding protein-1 (YB-1) is a member of the cold-shock domain (CSD) protein superfamily encoded by the *YBX1* gene. The N-terminal domain, the hydrophilic C-terminal domain, and the CSD are the main YB-1 domains [1]. Through its CSD, YB-1 interacts with inverted CCAAT boxes (Y-boxes) located on the promoter of many genes, such as DNA topoisomerase II alpha, proliferating cell nuclear antigen and multidrug resistance 1 [2], and determines the function. A wide variety of cellular functions, including DNA repair, gene transcription, mRNA splicing, translation, drug resistance, and stress responses to extracellular signals, are regulated by YB-1 [3,4]. YB-1, as reported in many human malignancies [4,5], is considered to be one of the most appropriate markers of malignant tumors and regulates cellular signaling pathways involved in nearly every cancer hallmark [4]. 

YB-1 can be phosphorylated at several serine residues. To exert the described functions, YB-1 must be phosphorylated at serine 102 (Ser-102) within the CSD. The expression statuses of YB-1 and Ser-102-phosphorylated YB-1 have been described to be reversely correlated with clinical outcomes, such as in diffuse large B-cell lymphoma patients [6]. Besides phosphorylation at Ser-102, it is also reported that phosphorylation of YB-1 at Ser-165 and Ser-176 sites activates NF-κB that leads to the regulation of NF-κB target genes and stimulation of proliferation in colon cancer cell lines [7,8]. 

YB-1 is generally expressed in the cytoplasm, but it is also transported to the nucleus at the end of G1-phase before S-phase entry [9] following cellular stress, such as anticancer agent treatment or hyperthermia [10,11], leading to therapy resistance [3,12]. The expression level of nuclear YB-1 has been described as a useful prognostic biomarker for poor overall survival and clinicopathological features in patients with colorectal cancer [13], prostate cancer [14], and non-small cell lung cancer (NSCLC) [15]. 

Ionizing radiation (IR) induces a variety of DNA damage, including single-strand breaks and double-strand breaks (DSBs). DSBs are the most lethal lesions and the main cause of radiotherapy-induced cell death. Radiation-induced DSBs are repaired by either homologous recombination or nonhomologous end-joining [16,17]. YB-1 participates in repair of damaged DNA, such as DSB repair [18] and base excision repair [19]. So far, the mechanism by which YB-1 stimulates DSB repair is not known. Function of YB-1 in repair of DSB can be a direct effect on DSB or an indirect effect. As an indirect effect, YB-1 has been described to have stimulatory effect on PARP1 as a nuclear protein with a high affinity for single-strand breaks as well as DSBs [20]. No direct effect of YB-1 on DSB repair has been reported so far. Independent of the mechanism of DSB repair by YB-1, YB-1 as a DNA damage response element needs to be present in the nucleus immediately after irradiation. This can occur through IR-induced translocation of phosphorylated YB-1 from the cytoplasm to the nucleus. Alternatively, activation of YB-1 in the nucleus can occur independent of its translocation from the cytoplasm to the nucleus. Our data indicate that phosphorylated YB-1 does not translocate to the nucleus. In fact, p90 ribosomal S6 kinase (RSK) translocates to the nucleus and stimulates already-existing YB-1 in the nucleus. 

## 2. Results

### 2.1. YB-1 Is Not Translocated to the Nucleus after Irradiation

**Figure 1:**
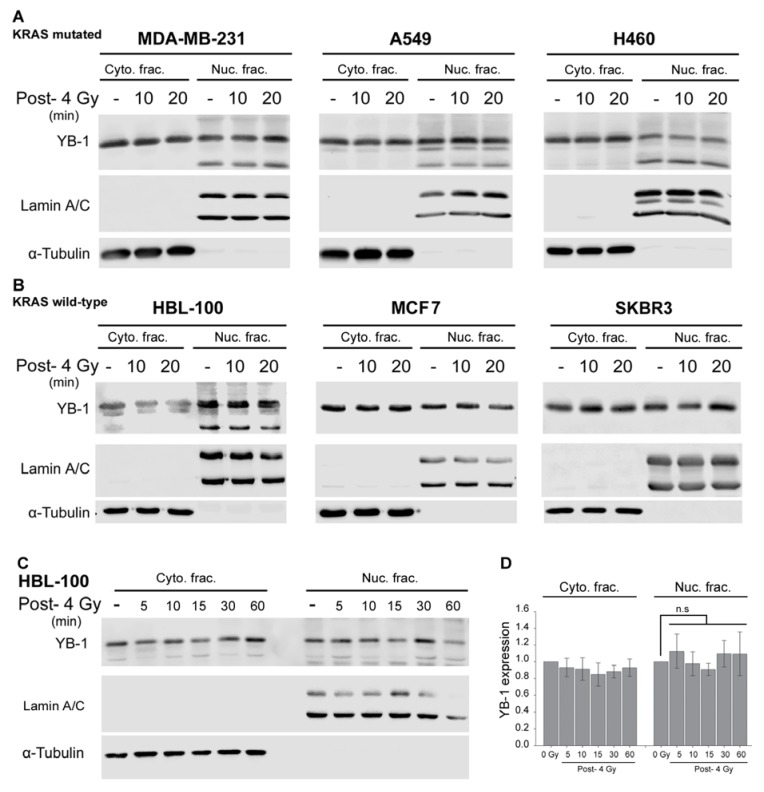
Y-box binding protein-1 (YB-1) is not translocated to the nucleus after irradiation. (**A**) A KRASmut breast cancer cell line (MDA-MB-231), non-small cell lung cancer cell lines (A549, H460), and (**B,C**) KRASwt breast cancer cell lines (HBL-100, MCF7, SKBR3) were irradiated (4 Gy). Cytoplasmic and nuclear protein fractions were isolated at the indicated time points after irradiation and subjected to SDS-PAGE. YB-1 was detected by Western blotting. α-Tubulin and lamin A/C were used as cytoplasmic and nuclear markers, respectively. (**D**) Histogram representing the densitometry data for the mean expression of YB-1 ± S.E.M. within one hour after ionizing radiation (IR) from four independent experiments in HBL-100 cells.

Previously, we demonstrated that exposure to clinically relevant doses of ionizing radiation induces phosphorylation of YB-1 in KRASwt but not in KRASmut breast cancer cells [18]. To date, it is not known whether acute phosphorylation of YB-1, such as occurs after irradiation, leads to nuclear accumulation of YB-1. Thus, expression of YB-1 in cytoplasmic and nuclear fractions was tested after irradiation in tumor cells from different origins, i.e., in the KRAS(G13D)-mutated breast cancer cell line MDA-MB-231, in the KRAS(Q61H)-mutated NSCLC cell line H460, and in the KRAS(G12V)-mutated NSCLC cell line A549. To investigate the impact of KRAS status on YB-1 nuclear accumulation, the KRASwt breast cancer cell lines MCF7, HBL-100, and SKBR3 as well as the head and neck squamous cell carcinoma (HNSCC) cell line FaDu were investigated. The data presented in Figure 1 indicate that exposure to ionizing radiation does not induce changes in the expression level of YB-1 within 20 min after irradiation in KRASmut (Figure 1A) or in KRASwt cells (Figure 1B). Similar results were obtained in HNSCC FaDu cells (data not shown). To investigate whether nuclear translocation of YB-1 takes longer than 20 min after irradiation, the pattern of YB-1 expression was studied in KRASwt HBL-100 cells within one hour after irradiation. The data shown again demonstrate that IR does not induce translocation of YB-1 to the nucleus within one hour post-irradiation (Figure 1C,D).

### 2.2. Phosphorylation of Nuclear YB-1 after Stimulation with EGF Is Associated with Nuclear Accumulation of RSK but Not of YB-1

**Figure 2:**
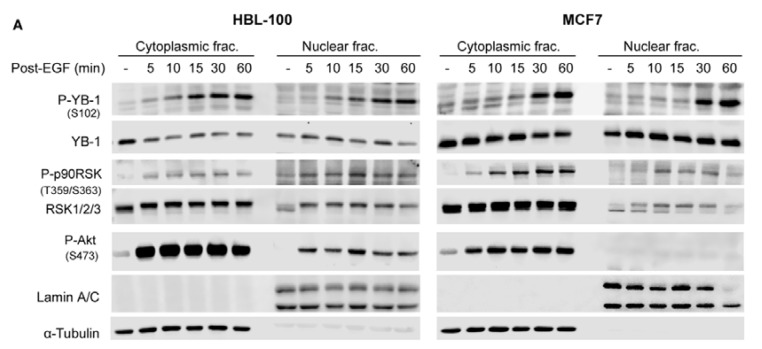

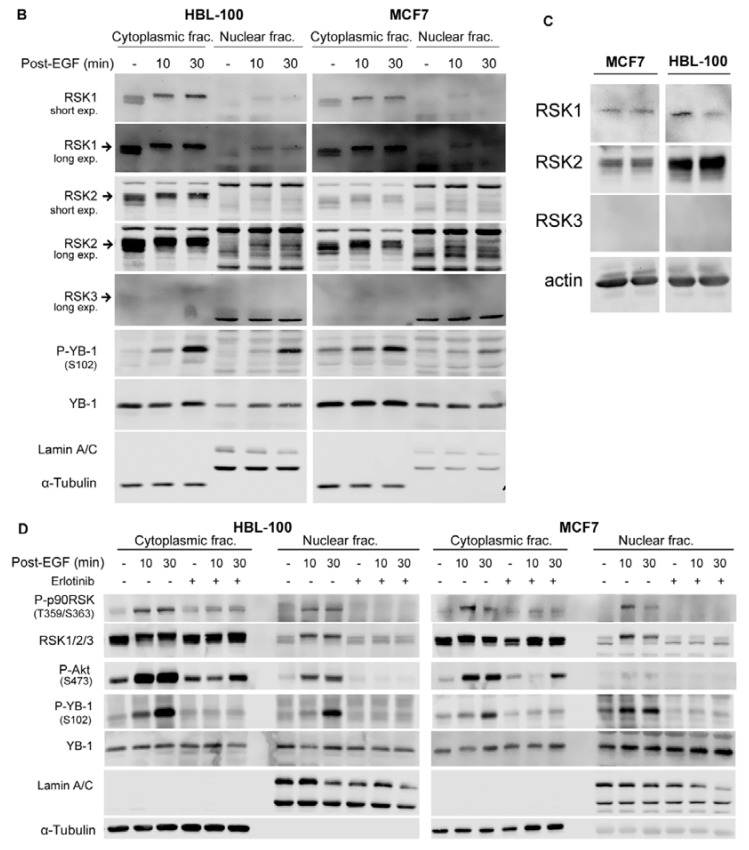
Phosphorylation of nuclear YB-1 after stimulation with epidermal growth factor (EGF) is associated with nuclear accumulation of RSK1 and RSK2 but not YB-1. (**A**,**B**) KRASwt HBL-100, and MCF7 cells were treated with EGF (100 ng/mL). Cytoplasmic and nuclear fractions were isolated at the indicated time points after stimulation. (**C**) Total protein samples (60 µg) were extracted under untreated control condition. (**D**) Cells were treated with erlotinib (10 µM, 2 h) followed by EGF treatment. Cytoplasmic and nuclear protein fractions were isolated at the 10 min and 30 min time-points after EGF treatment. P-RSK, RSK1/2/3, RSK1, RSK2, RSK3, P-YB-1, YB-1 and P-Akt were detected by Western botting. α-Tubulin and lamin A/C were used as cytoplasmic and nuclear markers, respectively.

Epidermal growth factor (EGF) induces YB-1 phosphorylation in KRASwt cells based on analysis of total cell lysates [18]. The data shown in Figure 1 demonstrate that the level of YB-1 remains constant within 20 min after irradiation. YB-1 stimulates repair of radiation-induced DSBs [18], a process that starts immediately after irradiation. Therefore, we investigated the expression status of nuclear YB-1 in KRAS wild-type HBL-100 cells and MCF7 cells within 1 h after stimulation with EGF. Treatment with EGF (100 ng/mL) led to phosphorylation of YB-1 in both the cytoplasmic and nuclear fractions in a time-dependent manner. RSK is the major kinase that regulates YB-1 phosphorylation [21,22]. Here, we show that EGF treatment stimulates RSK phosphorylation in both cytoplasmic and nuclear fractions in a time-independent manner. Interestingly, EGF treatment did not change the expression of nuclear YB-1 but enhanced the expression of nuclear RSK in both HBL-100 and MCF7 cells (Figure 2A). Interestingly, EGF treatment led to the nuclear translocation of RSK1 and RSK2 isoforms. RSK3 could not be detected in the cytoplasmic and nuclear fractions of both cell lines (Figure 2B). In a further experiment, by analyzing the expression of the RSK isoforms in total cell lysates we could show that RSK1 and RSK2 but not RSK3 are expressed in both HBL-100 and MCF7 cell lines (Figure 2C). 

Based on these data, we proposed that EGFR kinase activity stimulates accumulation of activated RSK in the nucleus, whereas RSK stimulates phosphorylation of nuclear YB-1. We investigated this hypothesis in cells pretreated with the EGFR tyrosine kinase inhibitor erlotinib (10 µM for 2 h) and those stimulated with EGF (100 ng/mL, 5 mins and 30 mins). The data presented in Figure 2D demonstrate that EGF induces phosphorylation of YB-1 and RSK in both cytoplasmic and nuclear fractions. Phosphorylation of Akt (Ser-473) was stimulated in the cytoplasmic fraction of HBL-100 and MCF7 cells. However, EGF induced Akt phosphorylation in the nuclear fraction of only HBL-100 cells. Interestingly, EGF induced translocation of RSK but not YB-1 to the nucleus. Erlotinib efficiently blocks phospho-RSK, translocation of RSK to the nucleus, and subsequent phosphorylation of YB-1 (Figure 2D).

### 2.3. Targeting RSK Inhibits Radiation-Induced YB-1 Phosphorylation in Nuclear Fractions without Affecting YB-1 Nuclear Expression

**Figure 3:**
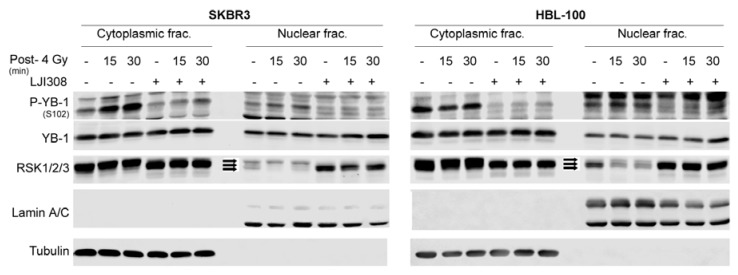
Targeting RSK inhibits radiation-induced YB-1 phosphorylation in nuclear fractions without affecting YB-1 nuclear level. SKBR3 and HBL-100 cells were treated with or without the RSK inhibitor LJI308 (both 5 µM, 2 h). At the indicated time-points after IR, cytoplasmic and nuclear protein fractions were prepared, and P-YB-1, YB-1, and RSK1/2/3 were detected by Western blotting. α-Tubulin and Lamin A/C were detected as cytoplasmic and nuclear markers, respectively. Arrows indicate distinct RSK isoforms.

Growth-factor-induced YB-1 phosphorylation in KRASwt cells [18] is mainly regulated through the MEK/RSK pathway [18,21]. In this study, we investigated the effect of RSK activity on phosphorylation of YB-1 in nuclear fractions after irradiation. In KRASwt SKBR3 cells, IR (4 Gy) induced YB-1 phosphorylation in both the cytoplasmic and nuclear fractions at 15 and 30 min post-irradiation. Pretreatment with the RSK inhibitor LJI308 (5 µM, 2 h) inhibited IR-induced phosphorylation of YB-1 in both fractions. Interestingly, in HBL-100 cells, radiation induced YB-1 phosphorylation only in the nuclear fraction at the 15 min time-point and was blocked by LJI308 (Figure 3). Again, these data indicate that IR-induced phosphorylation of nuclear YB-1 is not due to translocation of phospho-YB-1 from the cytoplasm to the nucleus. 

### 2.4. Conditional Expression of KRAS(G12V) Stimulates YB-1 Phosphorylation in Cytoplasmic and Nuclear Fractions without YB-1 Accumulation in the Nucleus

**Figure 4:**
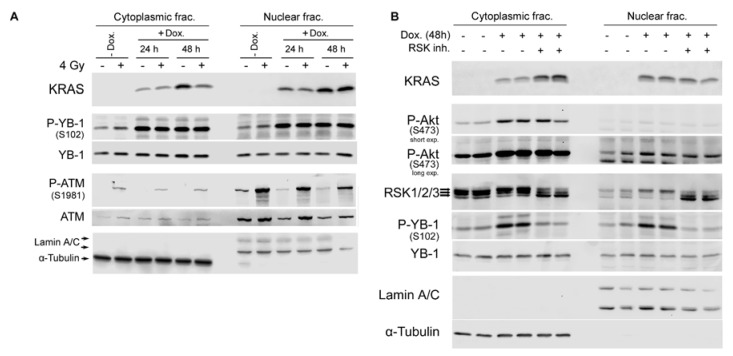
Conditional expression of KRAS(G12V) in the colorectal carcinoma cell line Caco2 induces YB-1 phosphorylation in the nucleus by stimulating RSK nuclear translocation. (**A**) Cells were treated with doxycycline (2 µg/mL) for the indicated times and mock irradiated or irradiated (4 Gy). Cytoplasmic and nuclear fractions were prepared 30 min after irradiation. (**B**) KRAS(G12V) was conditionally induced after treatment with doxycycline for 48 h. Treatment with the RSK inhibitor LJI308 (5 µM, 24 h) was performed followed by cytoplasmic and nuclear protein preparation. KRAS, P-YB-1, YB-1, P-ATM, ATM, and RSK1/2/3 levels were assessed via Western blotting. α-Tubulin and lamin A/C were detected as cytoplasmic and nuclear markers. Arrows indicate distinct RSK isoforms.

Exposure to ionizing radiation concomitantly induces YB-1 phosphorylation in both cytoplasmic and nuclear fractions of KRASwt cells. Thus far, the data presented demonstrate that phosphorylation of YB-1 in the nuclear fraction of KRASwt cells is not due to acute translocation of phospho-YB-1 or YB-1 to the nucleus. KRAS mutation leads to constitutive phosphorylation of YB-1 [18]. Here, we utilized Caco2 cells expressing an inducible KRAS(G12V) gene [23] to determine the effect of mutated KRAS on phosphorylation and localization of YB-1. KRAS expression became visible 24 h following doxycycline treatment and after 48 h was prominent in the total lysate as well as in the cytoplasmic fraction and, unexpectedly, in the nuclear fraction (Figure 4A). In the absence of doxycycline, IR (4 Gy) induced YB-1 phosphorylation in both cytoplasmic and nuclear fractions. Overexpression of KRAS(G12V) enhanced YB-1 phosphorylation in both fractions, which was not further enhanced by irradiation. Neither irradiation nor overexpression of KRAS(G12V) induced YB-1 accumulation in the nucleus. Phospho-ATM was detected as a DNA damage marker post-irradiation (Figure 4A).

The RSK family, important downstream effectors of the RAS/MAPK pathway, consists of four isoforms (RSK1–4) that regulate key cellular processes. We investigated whether RSK inhibition affects KRAS(G12V)-induced YB-1 phosphorylation in the nuclear fraction. The data shown in Figure 4B indicate that overexpression of KRAS(G12V) stimulates YB-1 phosphorylation in both fractions and that this effect was blocked by the RSK inhibitor LJI308 (5 µM, 24 h). Interestingly, although the RSK1–3 isoforms were expected to appear at 90 kDa, the RSK antibody against the three isoforms could distinguish distinct bands with slight differences in molecular weight. KRAS(G12V) expression only enhanced nuclear translocation of the RSK isoforms, presumably RSK2 as previously reported [24]. LJI308 blocked nuclear accumulation of the described RSK band but enhanced nuclear retention of the other isoform(s). The nuclear YB-1 level remained constant after KRAS(G12V) overexpression, independent of RSK activity status. 

## 3. Discussion

The activity and subcellular distribution of YB-1 are two important parameters for the function of YB-1. Currently, it is not known whether acute phosphorylation of YB-1 leads to its translocation to the nucleus, where it performs the majority of its functions. The importance of YB-1 localization to the nucleus is supported by the fact that the level of nuclear YB-1 is correlated with poor prognosis in various human cancers [5,13,14,15]. In the present study, we investigated the phosphorylation and expression patterns of nuclear YB-1 after IR, EGF treatment, and KRAS(G12V) overexpression. The data indicate that all the stimuli tested induced phosphorylation of nuclear YB-1 at Ser-102 without affecting its nuclear expression level as a consequence of nuclear translocation. The stimuli-induced phosphorylation of nuclear YB-1 at Ser-102 depends on RSK activity and is associated with nuclear translocation of RSK. 

YB-1 through its cold-shock domain interacts with double-stranded DNA [25]. Previously, we showed that exposure to IR induces phosphorylation of YB-1 at Ser-102, which stimulates repair of radiation-induced double-strand breaks (DSBs) [18]. YB-1 also protects cells from the cytotoxic effects of cisplatin, mitomycin C, UV light, and oxygen radicals [26]. The majority of IR-induced DSBs are repaired within the first 2–3 h post-irradiation by the fast component of DSB repair [16,27]. According to the function of YB-1 in DSB repair [18], nuclear localization of YB-1 is required for DNA repair in response to genotoxic stimuli, e.g., after exposure to IR. Sutherland et al. have reported that phosphorylation of YB-1 at Ser-102 by Akt is required for YB-1 nuclear localization in MCF7 breast cancer cells [28]. Since IR-induces YB-1 phosphorylation in KRASwt breast cancer cells, including MCF7 cells [18], it is expected that irradiation and other YB-1 stimuli, such as growth factors and KRAS mutation, will induce YB-1 nuclear translocation. In the current study, we did not observe marked nuclear localization of YB-1 after irradiation or EGF treatment, or following overexpression of KRAS(G12V) in tumor cells of different origins. Indeed, we observed high basal expression of YB-1 in the nuclear fractions of lung, breast, and head and neck cancer cell lines, which is in line with the data published by Basaki et al. in ovarian cancer cell lines [29]. Nuclear expression of YB-1 under non-stimulated conditions in the present study and the data reported by Basaki in ovarian cancer cells indicate that nuclear accumulation of YB-1 is a phenomenon that occurs gradually under basal stimulated conditions and, as reported, is regulated by Akt [28,29] and transportin-1 [30,31]. In the study by Basaki et al. [29], the authors tested whether stress-inducing exogenous addition of serum could stimulate nuclear translocation of YB-1 in seven serum-deprived human ovarian cancer cell lines. Among the seven cell lines, nuclear YB-1 translocation was not stimulated in five cell lines. Since nuclear translocation did not occur in the majority of cell lines tested, this observation is in line with our data, indicating the lack of YB-1 translocation in breast cancer cell lines after EGF treatment. IR, EGF, and KRAS(G12V) induce Akt phosphorylation [18] (also see Figure 2 and Figure 4). Interestingly, stimulation of EGFR by EGF induced phospho-Akt in the cytoplasmic fraction of both cell lines but only in the nuclear fraction of HBL-100 cells. Independent of this differential effect, YB-1 did not translocate to the nucleus in either of the cell lines. Thus, lack of YB-1 nuclear translocation after EGF treatment, irradiation, and KRAS(G12V) expression indicates that phosphorylation of Akt and YB-1 might be necessary but not sufficient for YB-1 translocation to the nucleus. Considering these data, we conclude that signaling cascades activated by EGF, IR, and KRAS(G12V) may directly lead to YB-1 phosphorylation in the nucleus. The conclusion for a direct phosphorylation of YB-1 in the nucleus without YB-1 nuclear translocation after cellular stress is mainly based on Western blot analyses of YB-1 in cytoplasmic and nuclear fractions. Although confocal microscopy analysis could strengthen our observation, we did not include the microscopy data due to the possible nonspecific binding of YB-1 antibodies used for immunohistochemical and immunofluorescent studies, which was reported by Wooley et al. [32].

**Figure 5:**
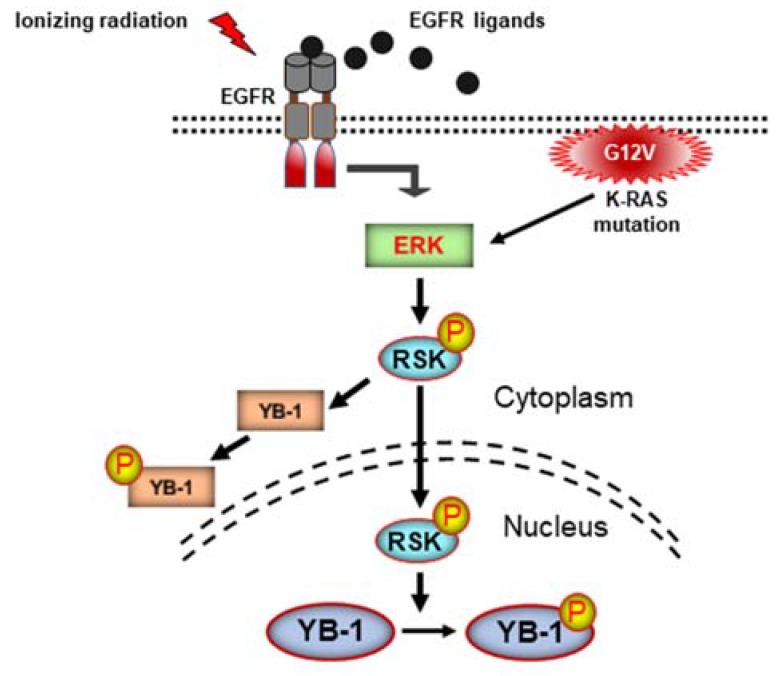
Schematic representation of the signaling cascade involved in Ser-102 phosphorylation of nuclear YB-1. Arrows indicate transition of YB-1 activating signals from the upstream stimulators.

RSK is the major kinase that phosphorylates YB-1 [21,22] by inhibiting the phosphatase activity of PP1 [24]. In the present study, we confirmed the role of RSK in YB-1 phosphorylation 1 at Ser-102 in the cytoplasmic and nuclear fractions by applying an RSK inhibitor. In addition to inhibition of YB-1 in the cytoplasm, RSK inhibition completely blocked YB-1 Ser-102 phosphorylation in the nucleus. Interestingly, inhibition of YB-1 phosphorylation was associated with inhibition of RSK translocation to the nucleus. The RSK family consists of four isoforms (RSK1–4) [33]. Among the different isoforms, RSK2 has been demonstrated to phosphorylate YB-1. In our study, the RSK antibody against 3 isoforms detected distinct bands, representing the different RSK isoforms. Stimulation of cells with EGF, irradiation, or KRAS(G12V) overexpression enhanced the translocation of one of the isoforms in association with enhanced YB-1 phosphorylation. According to the role of RSK2 in YB-1 phosphorylation [24], one of the RSK isoforms that accumulates in the nucleus and phosphorylates YB-1 is likely RSK2 (Figure 5). Investigating the status of RSK1, RSK2, and RSK3 revealed that besides RSK2, RSK1 is also translocated to the nucleus after EGF stimulation in two breast cancer cell lines tested. Whether different stimuli differentially affect subcellular distribution of the RSK isoforms and to which degree the specific RSK isoforms regulate phosphorylation of nuclear YB-1 are those issues that need to be investigated.

The signaling pathway described in Figure 5 is specifically depicted for the phosphorylation of YB-1 at Ser-102. Phosphorylation at Ser-165 and Ser-176 sites has also been shown to be crucial for YB-1 activity and YB-1-dependent activation of NF-κB, leading to the stimulation of cell proliferation in colon cancer [7,8]. Due to the lack of commercially available antibodies for the two described phosphorylation sites of YB-1, we were not able to analyze the effect of RSK activity on these two phosphorylation sites in the nucleus.

RAS GTPases, after mutation or external stimuli, localize to cell membranes for activation [34]. In the present study, we observed that KRAS(G12V) is also expressed in the nuclear fraction of Caco2 cells. This observation is in line with the report by Fuentes-Calvo et al., who showed the presence of KRAS-4B in fibroblast nuclei [35]. However, as also proposed by these authors [35], we cannot rule out a cross-reaction of the antibody used in our study with an undetermined nuclear protein that is also expressed after induction of KRAS(G12V). In this context, the selectivity and sensitivity of isoform- and mutation-specific RAS antibodies has been extensively investigated previously [36]. However, the antibody used in our work (Abcam, cat. # ab157255) was not tested in the study by Waters et al. [36]. While being cautious about the specificity of KRAS antibodies, we are investigating the possible function of nuclear KRAS(G12V).

In conclusion, as depicted in Figure 5, the acute appearance of phosphorylated nuclear YB-1 after EGF treatment, irradiation, and overexpression of KRAS(G12V) is not due to translocation of phospho-YB-1 or YB-1 from the cytoplasm to the nucleus. In fact, the described stimuli activate RSK, leading to its nuclear translocation, where it stimulates phosphorylation of already-existing nuclear YB-1. Our study adds crucial information to the very ambiguous mechanism of YB-1 activation in the nucleus. Moreover, the knowledge provided by this study can be implemented in the development of more appropriate and effective molecular targeting strategies for cancer to overcome therapy resistance and to improve patient survival.

## 4. Materials and Methods

### 4.1. Cell Culture

The KRAS (G13D)-mutated breast cancer cell line MDA-MB-231 (ATCC^®^ HTB-26^™^), KRAS(G12V)-mutated non-small cell lung cancer cell line A549 (ATCC, CCL-185^™^), KRAS (Q61H)-mutated non-small cell lung cancer cell line H460 (ATCC, HTB-177^™^), and KRASwt breast cancer cell lines MCF7 (ATCC^®^ HTB-22^™^), SKBR3 (ATCC^®^ HTB-30^™^), and HBL-100 (a spontaneously immortalized cell line that was obtained from primary cultures of cells derived from an early lactation sample of human milk [37]) were used in this study. Cells were cultured in DMEM (MDA-MB-231, A549), MEM (H460), or RPMI-1460 (SKBR3, HBL-100, MCF7) routinely supplemented with 10% fetal calf serum (FCS) and 1% penicillin–streptomycin. Additionally, we used the colorectal carcinoma cell line Caco2 stably transfected with doxycycline-inducible KRAS(G12V) [23], which allowed for conditional KRAS(G12V) expression. Caco2 cells were cultured in low-glucose DMEM (Lonza DMEM BE-12-707F, Cologne, Germany) supplemented with 10% *v*/*v* fetal bovine serum (FBS), penicillin (100 U/mL), streptomycin (100 μg/mL), puromycin (5 μg/mL), and blasticidin (5 μg/mL). All cells were incubated in a humidified atmosphere with 93% air and 7% CO_2_ at 37 °C.

### 4.2. Antibodies and Reagents

Antibodies against phospho-YB-1 (Ser-102) (cat. # 2900), YB-1 (cat. # 4202), phospho-p90RSK (cat. # 9344), RSK1/2/3 (cat. # 9355), RSK1 (cat. # 9333), RSK3 (cat. # 9343), P-Akt (Ser-473) (cat. # 4060), P-ATM (Ser-1981) (cat. # 4526), and ATM (cat. # 5883) were purchased from Cell Signaling (Frankfurt, Germany). RSK2 was purchased from ThermFisher Scientific (cat. # PA5-29280) (Karlsruhe, Germany). Antibodies against K-RAS (cat. # ab157255) and Lamin A/C (cat. # ab40567) were purchased from Abcam (Cambridge, UK). The anti α-tubulin antibody (cat. # CP06) was purchased from Calbiochem (Schwalbach, Germany). Epidermal growth factor (EGF) was purchased from Sigma-Aldrich (Taufkirchen, Germany). The EGFR inhibitor erlotinib was provided by Hoffmann-La Roche (Basel, Schweiz). LJI308 (cat. # S7871) was purchased from Selleckchem (Munich, Germany).

### 4.3. Irradiation, Ligand Stimulation, and Treatment with Inhibitors

Irradiation was performed at 37 °C using a Gulmay RS225 X-ray machine (Gulmay Limited, Chertsey, UK) at a dose rate of 1 Gy/min with the following exposure parameters: 200 kVp, 15 mA, and 0.5 mm copper additional filtering. At the indicated time points after EGF treatment (100 ng/mL) or irradiation, YB-1 phosphorylation was analyzed according to the design of each experiment. The inhibitors were dissolved in DMSO to create 10 mM stock solutions and stored at −20 °C. The inhibitor solutions were diluted in cell media to appropriate working concentrations just prior to use. Control cells received media that contained appropriate concentrations of the solvent DMSO.

### 4.4. Cellular Fractionation and Immunoblotting Analysis

Isolation of nuclear and cytoplasmic fractions was performed as previously described [38,39]. Total cell lysates were isolated as described previously [40]. Protein samples were subjected to sodium dodecyl sulfate polyacrylamide gel electrophoresis (SDS-PAGE). Following semi-dry blotting, membranes were incubated with specific antibodies. An ECL detection system was applied to visualize proteins using a LI-COR Biosciences system (Bad Homburg, Germany). α-Tubulin and Lamin A/C were detected as cytoplasmic and nuclear markers, respectively.

### 4.5. Densitometry and Statistics

Student’s *t*-test was performed to compare data between groups. A *p*-value less than 0.05 was considered to indicate a significant difference. Densitometric quantification analyses of immunoblots was performed using Image Studio Light Ver 5.2 from LI-COR Biosciences system (Bad Homburg, Germany).
